# Spatially uniform enhancement of single quantum dot emission using plasmonic grating decoupler

**DOI:** 10.1038/srep16796

**Published:** 2015-11-18

**Authors:** Arunandan Kumar, Jean-Claude Weeber, Alexandre Bouhelier, Fabien Eloi, Stéphanie Buil, Xavier Quélin, Michel Nasilowski, Benoit Dubertret, Jean-Pierre Hermier, Gérard Colas des Francs

**Affiliations:** 1 Laboratoire Interdisciplinaire Carnot de Bourgogne (ICB) UMR 6303 CNRS-Université Bourgogne Franche-Comté 9 Av. A. Savary, BP 47 870 F-21078 Dijon Cedex France; 2 Groupe d’étude de la Matière Condensée (GEMaC), UMR 8635 CNRS, UVSQ, Université Paris-Saclay, 45 avenue des Etats-Unis, 78035 Versailles Cedex, France; 3 Laboratoire de Physique et d’Etude des Matériaux, UMR8213 CNRS Ecole Supérieure de Physique et de Chimie Industrielles de la Ville de Paris, 10 Rue Vauquelin, 75231 Paris, France; 4 Institut Universitaire de France, 103, Bd Saint-Michel, 75005 Paris, France

## Abstract

We demonstrate a spatially uniform enhancement of *individual* quantum dot (QD) fluorescence emission using plasmonic grating decouplers on thin gold or silver films. Individual QDs are deposited within the grating in a controlled way to investigate the position dependency on both the radiation pattern and emission enhancement. We also describe the optimization of the grating decoupler. We achieve a fluorescence enhancement ~3 times higher than using flat plasmon film, for any QD position in the grating.

Future optical quantum devices require the development of photonic sources with control of light down to the single photon limit. Excellent examples of single photon emitters are the colloidal nanocrystal quantum dots (QDs) which are considered as the building blocks for future quantum devices such as quantum qubits and quantum cryptographic devices^1^,^2^. The application area of quantum emitters is wide and these applications require control of their emission such as emission rate, polarization, spectral properties, collection efficiency etc. Integration of single molecule or nanocrystals into plasmonic structures has recently proved to be one of the most promising yet challenging ways to control the emission properties at the single photon level^3^,^4^.

To date, various plasmonic structures have been investigated for the engineering of the spontaneous emission: nano-aperture^5^,^6^, random film^7^, plasmonic antenna^8^,^9^,^10^,^11^,^12^, circular grating^13^,^14^, in-plane cavity^15^, etc. All these devices relie on both the strong electromagnetic mode confinement that induces a large modification of the fluorescence emission rate and surface plasmon (de)coupled emission (SPCE)^16^ to improve the collection efficiency. However, photon decoupling and emission rate enhancement depend strongly on the emitter position within the plasmonic landscape. In this work, we are interested at integrating quantum emitter into plasmonic grating designed to achieve high fluorescence emission for any position of the QD in the grating.

## Results and Discussion

In this study, we use two different types of core-shell QDs. Purchased QDs (CdSeS/ZnS, from Sigma-Aldrich, diameter of 6 nm) presenting a thin shell will be referred as QD-1. Home-made CdSe/CdS QDs, synthesized following the ref. 4 present a thick shell of about 10 nm that leads to a suppression of the blinking (referred as QD-2 in the following)^17^,^18^,^19^. Both QD-1 and QD-2 present an emission spectrum centered at *λ*_*em*_ = 670 nm and with approximately 30 nm broadening (FWHM).

**Figure 1:**
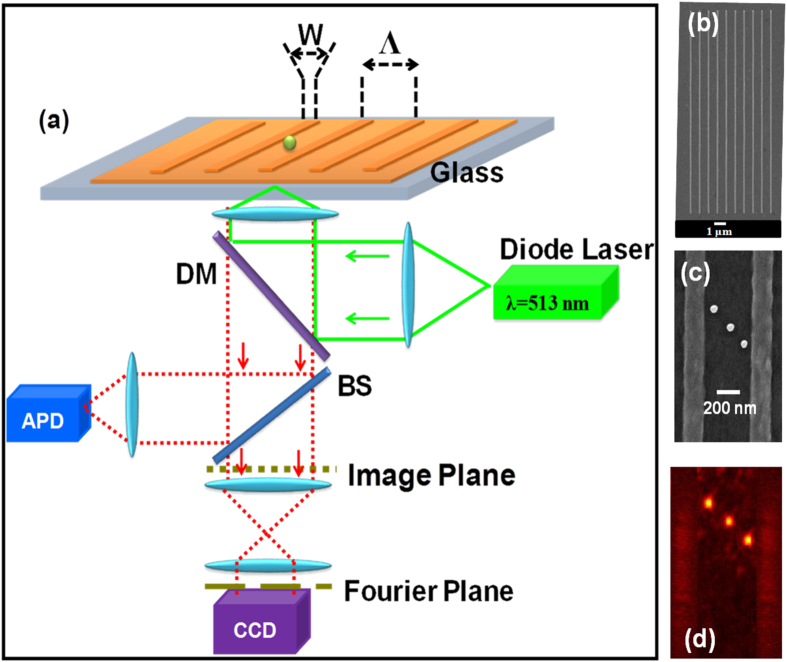
(**a**) Schematic description of the setup. (**b**) SEM of a gold plasmonic grating decoupler. (**c**) SEM image showing 3 individual QD-2 deposited between two ridges in a controlled way. (**d**) Fluorescence image of the individual QD-2 positioned into the grating decoupler in a controlled way.

To both enhance the fluorescence emission and maximize the collection efficiency, we consider plasmonic grating decoupler fabricated onto a continuous 50 nm metal film (gold or silver). The grating decoupler consists of ten nano-ridges with a period of *d* = 650 nm. This period is close to the effective wavelength of the surface plasmon polariton (SPP) propagating at the metal/air interface to ensure an efficient transfer momentum from the SPP towards an out-of-plane vertical direction (see Methods). Figure 1(b) presents a scanning electron micrograph (SEM) of a grating.

Since we are interested in the emission efficiency of QDs located within the grating, precise control of the QDs’ position is required. To this aim, we lithographied square boxes (100 nm × 100 nm) in a resist before deposition of QD and then remove the resist (see methods). Figure 1(c) shows the QD positioned between gratings with a precision of about 50 nm. Individual QD emission is recorded using a leakage radiation detection scheme (see Fig. 1(a) and ref. 15). QDs were imaged by using a confocal scanning microscope with an oil immersion 100X objective and numerical aperture (NA) of 1.3. QDs were excited at 513 nm wavelength with a cw laser diode. Figure 1(d) shows individual QD fluorescence emission within the grating. In the following, the position and the number of QDs is estimated from SEM images after each optical measurement.

### Fourier plane imaging and collection efficiency

**Figure 2:**
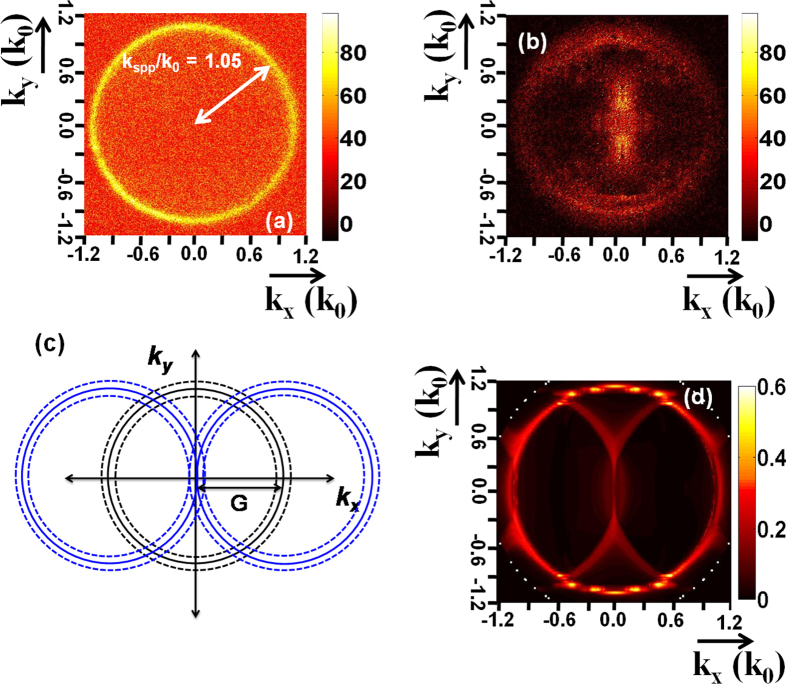
(**a**) Fourier space image of QD located on Au film showing a well defined momentum of *k*_*spp*_. (**b**) Fourier space image of QD emission when placed over a grating decoupler. (**c**) Schematic description of the Fourier image for QD decoupled emission. The solid lines correspond to the circle of radius *k*_*spp*_ (SPP mode excited by QD peak emission wavelength) and the dotted lines represent the broadening due to QD emission spectrum broadening. The central set of circles originate from the SPP directly excited at the Au-air interface. The off centered circles (center at ±*G*(= *k*_*spp*_) are due to the ±1 order diffraction by the grating. (**d**) Simulated Fourier image using FMM.

We first check the grating decoupling effect by imaging the emission diagram of the QDs by Fourier-plane leakage radiation microscopy (LRM)^15^,^20^. In order to obtain a sufficient signal to noise ratio (SNR) but keeping a controlled positioning of the QDs (determined by the size of the box), we considered ~5 QDs instead of single one deposited on the grating. Only thick-shell QD-2 were sufficiently bright to record a signal at this concentration level. We were not able to record any image in the Fourier plane using thin-shell purchased QD-1 due to a very low SNR (the home-made QD2 are brighter (about twice) than the purchased QD-1). Figure 2(a) presents the image recorded in the Fourier plane for QD-2 deposited on a bare gold film. The bright ring demonstrates isotropic surface plasmon coupled emission (SPCE) at the SPP momentum *k*_*SPP*_/*k*_0_ = 1.05 ± 0.02, in agreement with the calculated effective index (*n*_*SPP*_ = 1.04 ± 0.03). The calculated broadening Δ*n*_*SPP*_ = ±0.03 originates from the width of the emission spectrum (Δ*λ*_*em*_ = ±15 nm).

To see the effect of the grating decoupler on SPCE, Fourier space image were then measured on the SPP grating with approximately five QD-2 placed inside the grating as shown in Fig. 2(b). The intensity measured at the center of the Fourier plane reveals that the grating significantly modifies the angular emission by transferring the momentum of the propagating SPP^21^. The Fourier image presents two half-circles with a dominant intensity centered at 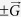, where 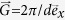 is the grating reciprocal wavevector (see also the scheme in Fig. 2(c). The maximum of intensity is therefore observed along the normal direction demonstrating the efficiency of the grating decoupler.

**Figure 3:**
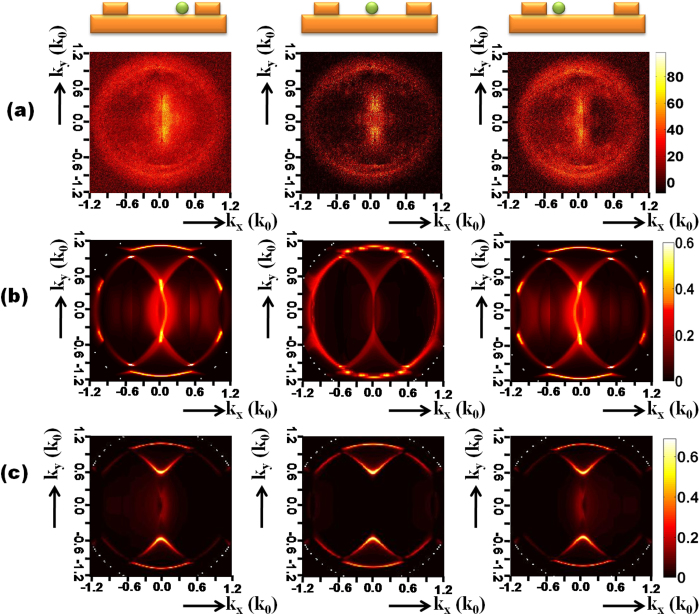
(**a**) LRM image recorded in the Fourier plane. (**b**) Calculated Fourier image taking into account the emission broadening [*λ*_*em*_ = (670 ± 15) nm]. (**c**) Calculated Fourier image at the peak emission wavelength (*λ*_*em*_ = 670 nm). The QD location is indicated on the top schemes.

Figure 2(d) represents the Fourier plane calculated employing Fourier modal method (FMM) following the work of Rigneault and coworkers^22^. The QD emission is modeled with 2 incoherent dipoles oriented perpendicular to the nanocrystal c-axis^23^. In order to reproduce the experimental situation, we have considered randomly oriented QDs and an emission wavelength broadening of 30 nm. The calculated Fourier plane (Fig. 2(d) is in very good agreement with the image recorded in the back-focal plane (Fig. 2(b)) and lets clearly appear the momentum transfer by the grating reciprocal wavevector as depicted in Fig. 2(c). Finally, for QDs deposited off-centered between two ridges, we observe similar Fourier plane but with some asymmetry as shown in Fig. 3(a). The light is slightly redirected in the direction of the closer ridge, in agreement with numerical simulations (Fig. 3(b)). For better understanding of the Fourier plane formation, we also present in Fig. 3(c) the images calculated at the QD emission peak (without any spectral broadening). For a centered QD, we observe a gap opening in the normal direction due to the interaction of the −1 and +1 orders diffracted SPCE and also small gap opening on the direct SPCE circle due to coupling between direct and diffracted SPCE^21^. The presence of light measured in the normal direction originates from the emission broadening. Last, we do not observe significative change in the Fourier plane images with the different width of the ridges (not shown).

Since the grating decoupler redirects SPCE within the light cone, it should significantly increase the collection efficiency of our setup. Table 1 presents the efficiencies calculated using FMM in reflection and transmission configurations. It is defined as the radiated power into the considered numerical aperture (NA), divided by the total radiated power for a randomly oriented nanocrystal. Due to numerical limitations, the dipolar emitter has to be located above the grating. Therefore, we compute the collection efficiency for a randomly oriented QD i)10 nm above a glass substrate, ii) 10 nm above an uncorrugated gold film or iii) 1 nm above the grating (i-e 51 nm above the gold film). Since the SPP extends over around 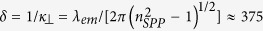 in air, we don’t expect a significant variation of the collection efficiency for QD 10 nm above the film, within the grating. For a QD deposited on a glass substrate, almost all the emitted light is scattered into the substrate so that nearly 70% of the emitted signal is collected through the glass, and only 5% is emitted in the air superstrate. A simple flat gold film slightly increases the collection efficiency in the upper medium due to a mirror effect but also decreases the collection efficiency by transmission. Note however that the full fluorescence signal may be increased compared to the bare substrate thanks to surface enhanced emission^24^. Finally, the grating decoupler strongly increases the collection efficiency in the superstrate since the SPCE is decoupled into the light cone. The collection efficiencies in the superstrate and in the substrate are comparable. This demonstrates the efficiency of the grating decoupler. The collected signal into air can be further increased using optically thick film, mitigating thus the need of expensive immersion objectives.

### Radiative decay rate and apparent quantum yield

The grating decoupler modifies the radiative rate by opening new decay channels within the light cone in air. To check this, we estimate the total and radiative decay rates using the Green’s dyad formalism^15^,^25^,^26^,^27^. The total decay rate is exactly calculated considering the emitted power for a 2D-degenerated dipole perpendicular to the crystal c-axis^23^. The radiative decay rate is approximated assuming a lossless system. See methods for more details.

**Figure 4:**
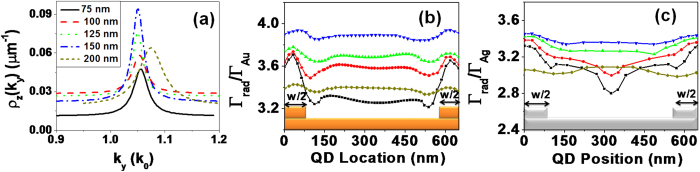
(**a**) 2D-LDOS calculated inside a gold grating for different widths of the ridges. LDOS is shown in the range of wave vectors from 0.9 to 1.2 where the dominant peak due to coupling to SPPs observed. (**b**,**c**) Calculated radiative decay rate enhancement as a function of position for randomly oriented QD inside the cavity for different gold (**b**) or silver (**c**) SPP grating width *w*. Radiative decay rates are normalized with respect to the radiative decay rate obtained on a bare metal film.

The grating is modeled by means of five ridges given that more periods do not change significantly the achieved results. In Fig. 4(a), we present the 2D-LDOS for a vertically oriented dipole calculated for the different ridge widths considered in this work. It presents a resonant behaviour, typical from coupling to a mode, here a SPP at effective index *n*_*SPP*_ = 1.05. We also observe that the effective index is shifted for large ridges (*w* = 200 nm) since the high duty cycle of the grating modifies the properties of the Bloch mode supported by the grating. Therefore, the grating decoupler period has to be modified accordingly for large duty cycle, as pointed out in ref. 28,29. Finally, the 2D-LDOS presents a maximum for a ridge width of 150 nm, indicating an optimum coupling between the QD emission and the grating.

Furthermore, we calculate the radiative rate as a function of the position within the grating in Fig. 4(b). For simplicity, it is averaged over the orientations of the QD. The radiative decay rate is normalized with respect to the decay rate over a flat gold film to outline the effect of the grating. The QD radiative rate increases inside the gratings with respect to the radiative rate above a flat gold film up to a factor of nearly four for the optimum ridges width. The small asymmetry of the decay rate with respect to the center originates from the finite and odd number of ridges. Note also that the silver grating decoupler leads to similar enhancement values with respect to the flat silver film. This however corresponds to higher effect compared to free-space emission since the SPP supported by a silver film presents lower losses than a gold film.

Importantly, we observe that the radiative decay rate presents significative spatial variations for either gold or silver gratings, except for the optimum width *w* = 150 nm. This property is even more visible for a silver grating as shown in Fig. 4(c). We attribute the strong depletion of the radiative rate in the silver grating for *w* = 75 nm but hardly visible for the gold grating to a different phase shift at reflexion on silver and gold ridges. For *w* = 150 nm, the radiative rate is practically independent of the position. Close inspection of the numerical simulations reveals that the averaging over the QD orientation slightly flattens the position dependency but does not explain the effect of the ridges width. We therefore attribute this effect to the change of the length of the cavity that is formed between two ridges. Indeed, the cavity length (from ridges side to side) varies from 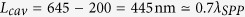 for *w* = 200 nm to 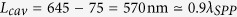 for *w* = 75 nm. We expect a significant enhancement of the decay rate for cavity length *L*_*cav*_ = *nλ*_*SPP*_/2^15^,^30^. For the intermediate case 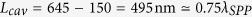 we observe only weak dependance of the decay rate on the position into the cavity. Finally, the optimum grating combines efficient decoupling efficiency (period of the grating decoupler close to *λ*_*SPP*_) and cavity effect between two ridges (width of the ridges such that 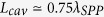).

### Fluorescence enhancement

**Figure 5:**
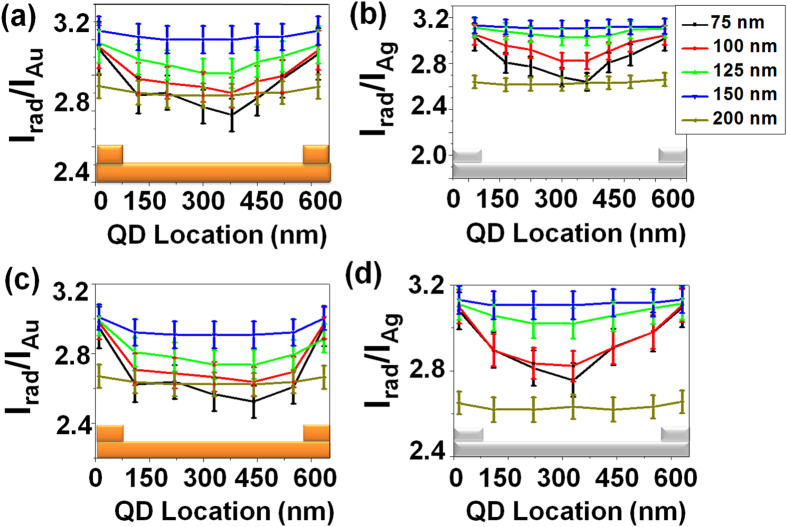
QD emission intensity enhancement on SPP device as compared to flat Au or Ag surface with different width of ridges and at different positions inside the sample for (a,c) gold and (b,d) silver. (**a**,**b**) are for QD-1 and (**c**,**d**) for QD-2. Error bars give the standard deviation estimated from successive (~70) individual measurements for each QD position. Legend colors are the same as for Fig. 4.

To experimentally investigate this behaviour, we measured the fluorescence signal of single QD deposited inside grating decouplers. The signal enhancement inside the grating is given with respect to a QD placed on flat gold (or silver) film on the same sample. We considered emission for either (purchased thin shell) QD-1 or (home synthesized thick shell) QD-2. We observe an increase of the fluorescence emission as shown in Fig. 5. The intensity enhancement is nearly similar for both types of samples (gold or silver) and QDs (commercial or home made) when compared to a flat metal film. Since the measured intensity enhancements are similar for QD-1 and QD-2, we conclude that the thick shell of QD-2 acts as the spacing layer and avoids a step of depositing a spacer layer as in case of QD-1. Moreover, the intensity enhancement is found to be dependent on the width of nano-ridges and presents a behaviour very similar to the calculated radiative rate (Fig. 4). We discuss this behaviour in details below.

The fluorescence enhancement can originate from the enhancement of the excitation field and the emission rate. To estimate the excitation enhancement, we compare the excitation intensity in the grating (*I*_*grating*_) and on a flat metal film (*I*_*slab*_). For gold, we calculate *I*_*grating*_/*I*_*slab*_ = 1.3 at *λ* = 513 nm (see Methods). The electric field at the QD position is governed by the Au/air SPP that is excited at oblique incidence for the flat field and close to the normal incidence in presence of the grating coupler. To estimate the emission enhancement, we proceed as follows. We calculate the apparent quantum yield inside the grating and on the metal slab. The apparent quantum yield is defined as the ratio between the radiative and total rates in the structure. We found *η*^*grating*^/*η*^*slab*^ ≈ 3 so that the fluorescence enhancement due to the grating is estimated to 1.3 × 3 = 3.9 for *w* = 150 nm, in agreement with our data. We achieve a similar value for the silver grating. Our simulations indicate that the main difference between the bare metal film and the grating is the radiative contribution. Indeed, the non radiative rate (that includes absorption and non radiative energy transfer) dominates the QD relaxation near a flat film (*η*^*slab*^ ≈ 30%) whereas the grating decouples almost all the energy into the radiative rate (*η*^*grating*^ ≈ 90%). As a consequence, the fluorescence enhancement is mainly governed by the radiative rate and is weakly affected by the excitation field enhancement. That is why we observe a similar behaviour for the fluorescence enhancement measured in Fig. 5 and the radiative rate calculated in Fig. 4.

Next, we focus on the spatial variation of the intensity enhancement inside the grating (see Fig. 5). For grating made of ridges of width *w* = 75 nm, the measured signal depends on the position of QDs inside the grating. For the optimum width *w* = 150 nm, it is however constant on the full position range. These results show that the SPP device with grating of 150 nm width is optimum in terms of both intensity enhancement and spatial invariance. Similar results are observed for the Ag samples and/or QD-2. The similarity in the results of gold and silver samples can be attributed to the almost equivalent effective indices for SPPs propagating at Au-Air and Ag-Air interfaces.

**Figure 6:**
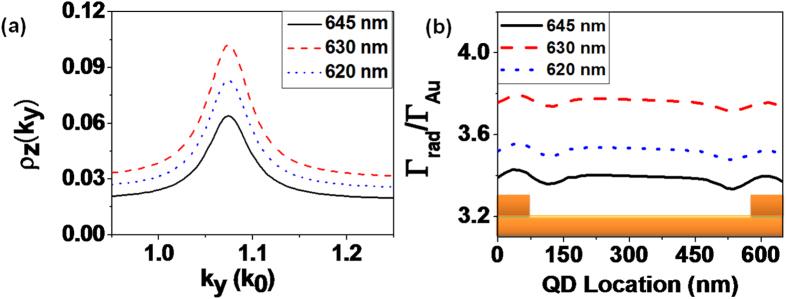
Calculated density of states for SPP grating having 200 nm width of gold ridges and different periods. (**b**) Calculated enhancement in radiative decay rate as a function of position of the QD inside the cavity for the corresponding grating decouplers.

In addition, it can be observed from Fig. 5 that the coupling rate decreases after increasing the nano-ridge width to 200 nm due to the mismatch between the grating period and the supported mode for this filling factor (see Fig. 4(a)). We therefore design a new grating to study this effect. Figure 6(a) presents the 2D-LDOS as a function of *k*_*y*_ calculated for ridges of width *w* = 200 nm for different grating periods. The optimum period for this filling factor is *d* = 630 nm, that corresponds to a mode of effective index *n*_*eff*_ = 1.06 in agreement with the LDOS calculated in Fig. 4(a) for *d* = 650 nm and *w* = 200 nm. This again demonstrates the influence of the ridges size on the grating design^28^. The radiative rate is calculated for 200 nm nano-ridges for gold samples to clearly observe this effect, see Fig. 6(b). Radiative rate is highest for the 630 nm period.

**Figure 7:**
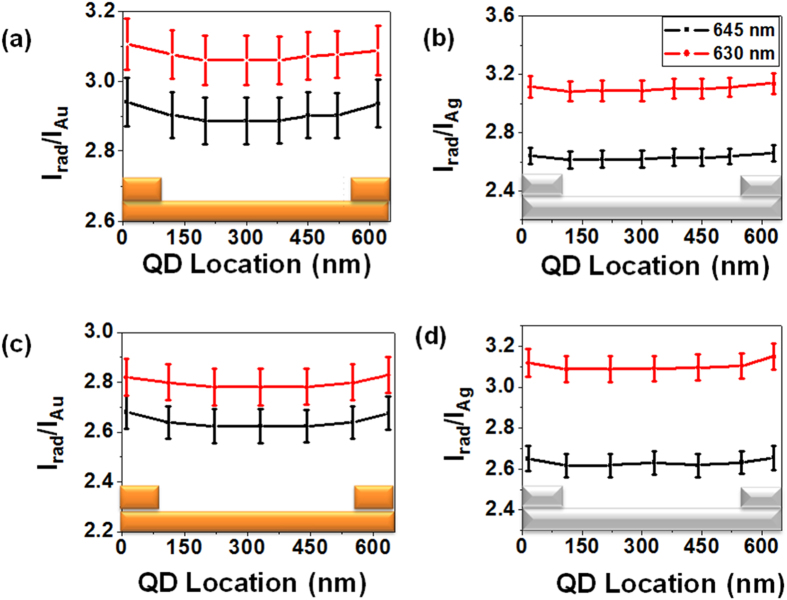
QD intensity enhancement for SPP device with ridge widths of 200 nm for reduced grating periods showing the enhanced emission rates for reduced grating periods. (**a**,**c**) are for the samples with gold and (**b**,**d**) for silver. (**a**,**b**) are for QD-1 and (**c**,**d**) for QD-2.

To confirm this observation, the QD intensities were measured on Au and Ag SPP devices with ridges of 200 nm width and a grating period of 630 nm for both QD-1 and QD-2. Figure 7 depicts the measured QD intensity enhancement. For reference, this figure also shows the enhancement ratio for grating with 645 nm periods (see also Fig. 5) and clearly demonstrates the enhancement of emission for QDs located at all positions. This confirms that the fluorescence enhancement is mainly governed by the radiative rate (compare Figs 7 and 6(b)).

## Conclusion

To summarize, we have demonstrated that grating permits strong control of the fluorescence signal at the single QD level. Grating efficiently decouples the SPPs at Au (Ag)-air interface excited by the QD in the normal direction. Moreover, the optimization of the grating width provides an optimum enhancement for all positions inside the grating. The two important parameters are the grating period and duty cycle. The period should be close to the SPP wavelength *λ*_*SPP*_ to ensure efficient decoupling of the QD emission (with an exact value that slightly depends on the grating duty cycle). The duty cycle of the grating is mainly determined by the length of the cavity formed between two ridges that should be close to 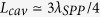. We achieve an enhancement of the QD emission of about 3 everywhere in the sample so that no controlled positionning of the QDs is needed for future experiments. Moreover, we have chosen straight grating in order to fully characterize the role of each parameter. These results should be extended to circular gratings with potentially stronger effect. The control of QD emission, namely both the collection efficiency and the intensity enhancement, without strong constraint on their positionning, is promissing for quantum devices applications.

## Methods

### Samples fabrication

Samples were fabricated using electron-beam lithography. The grating was first designed on ITO glass substrates by e-beam lithography before thermal evaporation of 50 nm of metal. The samples were then covered by a 50 nm thick metal film conformed on the 50 nm metal gratings. We designed different sets of gratings. The grating decoupler consists of ten nano-ridges of varying width (75, 100, 125, 150 and 200 nm), 50 nm height and with a period *d* = 650 nm. Indeed, the grating leads to momentum transfer between the surface and diffracted waves such that 
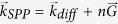
 *i.–e.* 
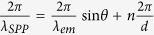
 where n and *θ* are the diffracted order and angle, respectively. The grating period *d* ≈ *λ*_*SPP*_ ensures efficient (de)coupling of the surface wave to the 1^*st*^ diffracted wave at the normal direction. The Au/air and Ag/air SPP wavelengths are *λ*_*SPP*_ = 645 nm (effective index 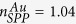) and *λ*_*SPP*_ = 653 nm 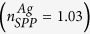 respectively, for a glass/metal (50 nm)/air slab at the QDs emission wavelength *λ*_*em*_ = 670 nm so that we fixed *d* = 650 nm.

For QD-1, an additional 5 nm thick SiOx layer was deposited by thermal evaporation to avoid fluorescence quenching by non radiative energy transfer to the metal film. For QD-2 no additional spacing layer is required, owing to their thick shell.

In order to position the QD in the sample in a controlled way, we proceed as follows. A poly(methyl methacrylate) (PMMA) film was spin coated onto the sample into which square boxes (100 nm × 100 nm) were lithographied. Then, a diluted QD solution was drop casted. Afterwards, the PMMA resist was removed from the samples by immersion into acetone for 5 minutes.

### Computation of the decay rates

The decay rate of QD is calculated according to the classical description of a dipolar emitter. First, we calculate the 2D-local density of states 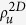 within the grating decoupler. 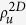 is related to the power dissipated by an oscillating unitary dipole **u** at a given in-plane wave vector *k*_*y*_ along the invariant y-axis. It expresses as a function of the 2D-Green’s dyad associated to the plasmonic grating^25^ 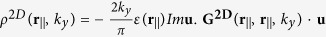. In this expression, 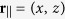 is the dipole position in the transverse plane, and the 2D-Green’s dyad is numerically computed as detailled in ref. 25. The total decay rate of a dipolar emitter is then determined from a numerical integration^26^ 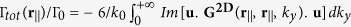. Finally, the decay rate associated to the 2D-degenerated dipole of the QD nanoscrystal is the average of the decay rates of the two (incoherent) single dipoles. The dielectric constant of gold and silver are taken from tabulated data^31^.

The radiative decay rate is calculated as the power scattered in the radiative zone and is achieved using the Fourier modal method (FMM) but only for a dipole above the grating due to numerical difficulties for a source located between two ridges. Since the radiative rate strongly depends on the dipole height above the metal film, we also estimated this quantity from the Green’s dyad technique (GDT). To this aim, we computed the total decay rate as described above but assuming a lossless system (namely, we fixed the imaginary part of the dielectric constant of the metal to zero). We have compared the exact radiative rate obtained using the FMM and approximated using GDT for a lossless system both above a flat metal film and above a plasmonic grating. We have estimated that the error made on the radiative rate approximated using the Green’s dyad Technique on the lossless system is less than 15%.

### Estimation of the fluorescence enhancement

The enhancement of the fluorescence signal originates from the increase of both i) the excitation rate and ii) quantum yield in presence of the grating. Using FMM, we estimate the electric field enhancement 10 nm above the gold film, assuming an excitation from the substrate with a NA = 1.3 (*λ*_*X*_ = 513 nm). For a flat gold film, we achieve *I*_*slab*_ = 6.7*I*_0_ that is mainly due to the excitation of a SPP at the angle of incidence *θ*_*inc*_ = 43.4° such that *n*_*SPP*_ = *n*_*glass*_*sinθ*_*inc*_ (*I*_0_ is the incident intensity). The enhancement slightly increases to *I*_*grating*_ = 8.7*I*_0_ inside the gold grating with a major contribution of the excitation close to the normal incidence, as expected for a grating coupler (*θ*_*inc*_ = 13.9°, in agreement with 
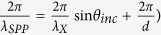
. Eventually, when compared to the flat gold film, we achieve an enhancement of the excitation rate due to the grating of *I*_*grating*_/*I*_*slab*_ = 1.3. The apparent quantum yield, defined as the ratio *η* = Γ_*rad*_/Γ_*tot*_, is calculated using GDT as discussed above. Finally, the fluorescent signal is estimated from *I*_*fluo*_ ∝ *σ*_*abs*_*I*_*SPP*_ × *η*, where *σ*_*abs*_ is the absorption cross-section of QDs.

## Additional Information

**How to cite this article**: Kumar, A. *et al.* Spatially uniform enhancement of single quantum dot emission using plasmonic grating decoupler. *Sci. Rep.* **5**, 16796; doi: 10.1038/srep16796 (2015).

## Figures and Tables

**Table 1:** Collection efficiencies calculated with a detection from the superstrate using a objective with NA = 0.6 and with a detection from the glass substrate with a objective opened at 1.3.

	glass substrate	gold film	grating decoupler^a^
*η*_*refl*_ (NA = 0.6)	4.5%	7.6%	23%
*η*_*trans*_ (NA = 1.3)	68%	54%	26%

The emitter is placed 10 nm above a glass substrate or a 50 nm gold film. The emission wavelength is *λ* = 670 nm.

^a^QD 1 nm above the grating, centered between 2 ridges.
